# Sharing believable stories: A qualitative study exploring the relevance of case studies for influencing the creation of healthy environments

**DOI:** 10.1016/j.healthplace.2021.102615

**Published:** 2021-09

**Authors:** Anna Le Gouais, Louise Foley, David Ogilvie, Jenna Panter, Cornelia Guell

**Affiliations:** a MRC Epidemiology Unit & Centre for Diet and Activity Research (CEDAR), School of Clinical Medicine, University of Cambridge, Box 285 Institute of Metabolic Science, Cambridge Biomedical Campus, Cambridge, CB2 0QQ, UK; b European Centre for Environment and Human Health, University of Exeter Medical School, Truro, TR1 3HD, UK

**Keywords:** Case studies, Environmental determinants of health, Decision-making, Place-making, Qualitative

## Abstract

Case study examples can inform policy recommendations and action to create healthy environments. This qualitative study, using semi-structured interviews with nine cross-sectoral stakeholders in England, explored the role of context in case study examples. We found that case studies can not only be a ‘practical example’ but also used as a ‘believable story’ with the power to influence decision-making. Case studies may be deemed believable if similar and locally relevant, but judgements can be inherently political and politicised. Metrics used to measure case study outcomes can differ in believability. Storytellers who understand different audiences can be used to build support.

## Highlights

• Case study examples can be used to tell believable stories to influence decision-making.
• Believable stories are ways of organising and framing multiple pieces of evidence.
• Physical, socio-economic and political issues will affect believability.
• The type of data used to demonstrate impact should vary depending on the audience.
• Believable stories may increase acceptability of environmental change.

## 1 Introduction

Environmental change can be more influential and equitable than individual behaviour change strategies to tackle population health issues such as physical inactivity (Adams et al., 2016; Wu et al., 2011). Often decision-makers from non-health sectors such as transport, urban planning and housing have the power to change these environmental determinants of health. These decision-makers tend to value precedent and case study examples to guide ‘what works’ (Guell et al., 2017; Le Gouais et al., 2020a,b; Lge-Elegbede et al., 2020). Yet, ‘what works’ in other settings may also highlight differences between settings and call into question the transferability of case study examples. Equally, evidence reviews, trials and interventions are increasingly critiqued for their generalisability, demanding to clarify ‘what works, for whom, in what circumstances’ (Pawson and Tilley, 1997). Challenging such views, Ogilvie et al. (2020) argue that it is better to look beyond the particular form of interventions that may be place-specific, such as a cycle lane of a certain physical type, to understand their underlying and potentially more generalisable functions, such as altering connectivity.

Contextual features of case study examples may influence whether decision-makers choose to replicate or adapt environmental measures from elsewhere. However, context can involve many different aspects, including physical, cultural, social, economic, historical, and political features (Craig et al., 2018). Greater clarity on the perceived importance of particular types of contextual features associated with place-based interventions that may affect applicability (the extent to which an intervention may be implemented in another setting (Burchett et al., 2013; Wang et al., 2006)) and transferability (the extent that the measured effectiveness is applicable in another specific setting (Wang et al., 2006)) could be useful. This could help public health researchers who plan, evaluate and publish findings of complex interventions. It could also be useful for knowledge brokers who share information and evidence (Choi et al., 2005; Kingdon, 2010; Sallis et al., 2016) – because emotions and values are known to be important in decision-making (Cairney and Kwiatkowski, 2017; Cairney and Oliver, 2017; Knaggård, 2015), understanding how to leverage case studies to persuade decision-makers of the value of environmental change for population health benefit is important. Finally, clearer understanding about contextual relevance could also help shape future guidance material to support stakeholders from different sectors to create healthier environments, such as more effective walking and cycling infrastructure.

This study aimed to understand what contextual factors are viewed as important by local stakeholders when influencing creation of healthy environments and decisions that affect environmental determinants of health. We investigated this with a qualitative study to understand stakeholders’ perceptions of context for case study examples of new walking and cycling routes, which may influence levels of physical activity and population health. The research was guided by two main questions: (1) In what ways do case studies shape decision-making? (2) What contextual issues are most important to decision-makers in order that case-study examples of walking and cycling routes appear relevant?

## 2 Methods

### 2.1 Study setting and participants

The study focused on two purposively selected local government areas in England and private sector developers that were involved in large mixed-use developments in those areas. These were contextually different in terms of deprivation, urbanisation, topography, and levels of cycling. Both areas had public health practitioners with a dedicated urban planning role. Area 1 was a relatively wealthy semi-rural district with major growth areas; Area 2 included a deprived urban area undergoing regeneration.

Eight semi-structured interviews were conducted with nine stakeholders purposively sampled to include public health (with an urban planning focus), urban planning, transport planning (including walking and cycling infrastructure), elected councillors and private sector developers. Interviewees were selected from participants of a previous study investigating decision-making for new walking and cycling infrastructure and open spaces (Le Gouais et al., 2020a) which recruited 40 participants across three areas of England using snowball sampling (Patton, 2002). They were deemed to be information-rich because of their roles and experience. The councillors each came from different political parties. The private sector participants were recruited for the previous study because they worked on a large-scale mixed-use development in one of the focal areas, however, their experiences spanned multiple areas.

Where short-listed previous study participants were no longer contactable (n = 3), alternatives were invited through snowball sampling (Patton, 2002). These are shown in Table 1. Three people declined to take part because they were too busy (one public health practitioner, one local government urban planner and one private sector urban planner). The two participants not included in the original study replaced individuals who had changed roles. They had previously been in contact with ALG and sent information about the original study and its findings.

**Table 1:** Summary of study participants.

Sector	Area 1	Area 2	Private sector	Total
Public Health	1			1
Urban planning	1^ab^	1^c^	1	3
Transport planning	1^a^		1	2
Councillor	2 (district^b^ and county)	1		3
TOTAL	5	2	2	9

a Interviewed together.

b Not included in the original study.

c Changed organisations.

### 2.2 Data collection

**Fig. 1:**
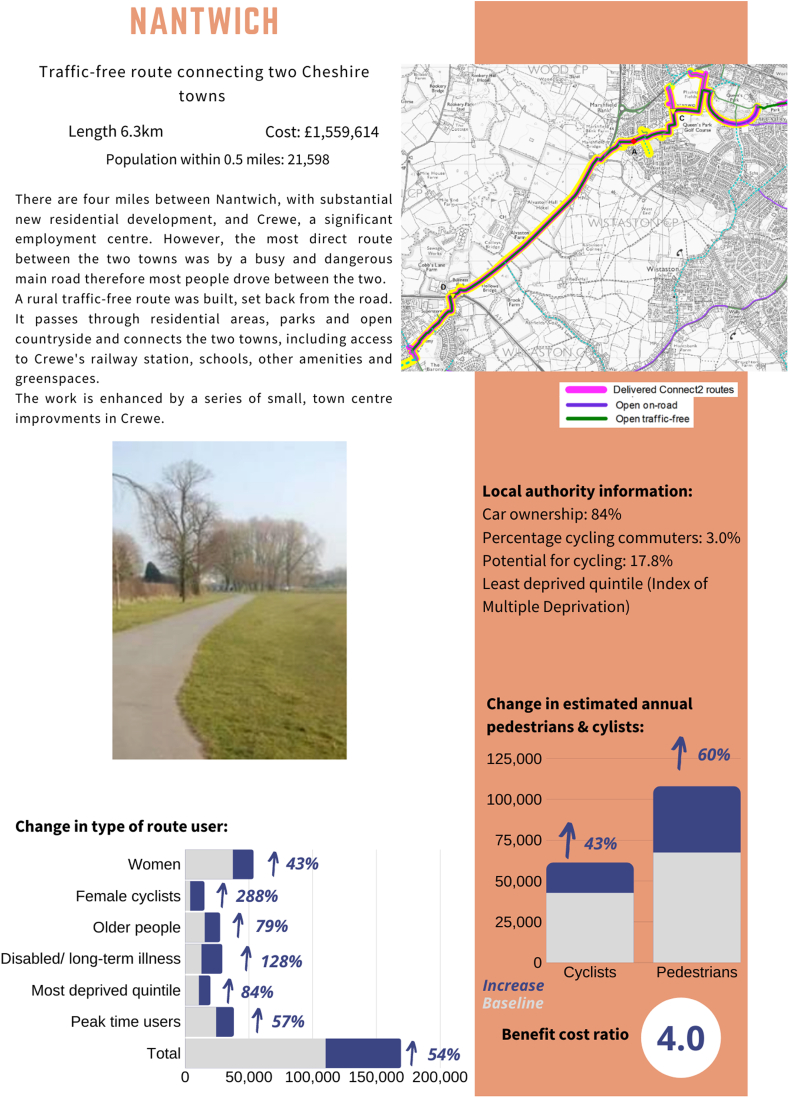
Case study example interview prompt sheet.

**Fig. 2:**
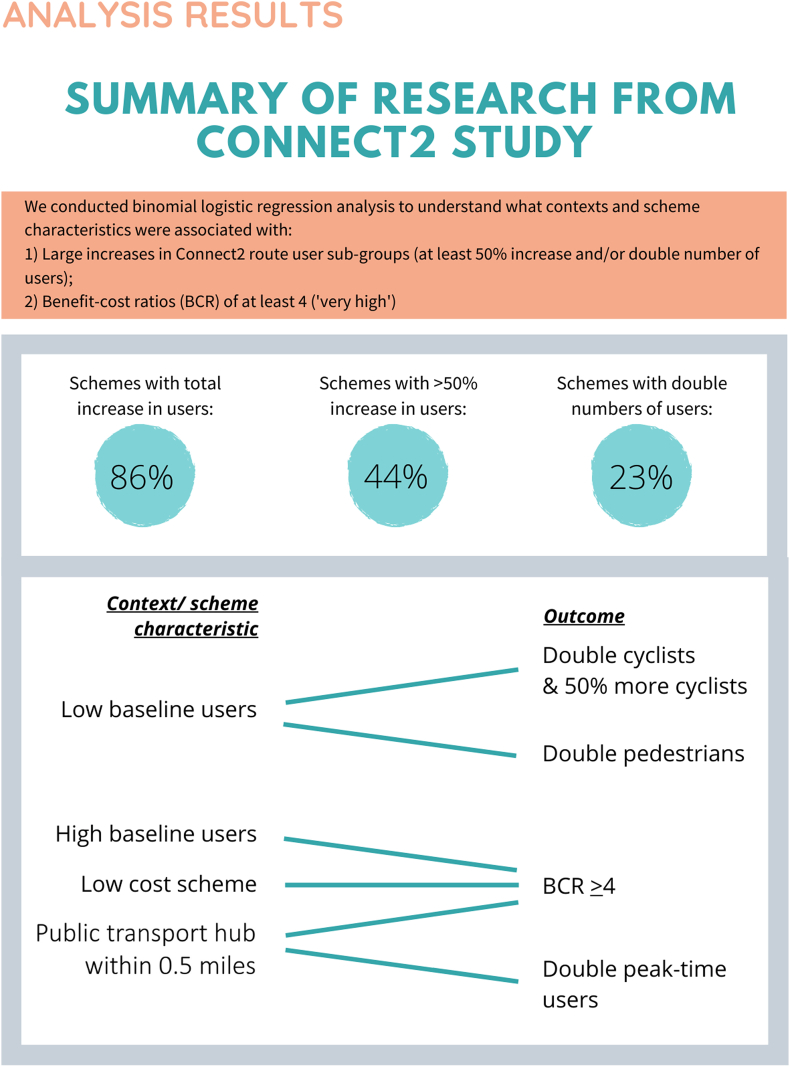
Connect2 results infographic interview prompt sheet.

We used semi-structured interviews to gain in-depth insights into perceptions of context for case studies of new walking and cycling routes. As part of formative work to improve the relevance of the current study, we conducted content analysis of data from a previous qualitative study (published elsewhere (Le Gouais et al., 2020a)) to identify gaps in understanding and knowledge amongst stakeholders, followed by a short questionnaire to check level of interest in these topics, as well as to understand in what format information was preferred. Feedback was obtained from 56% of people (n = 43). We identified strong interest in understanding more about use of new walking and cycling routes, economic impacts of walking and cycling routes, and examples of good cycling infrastructure and other active living infrastructure (ALI) designs. The most popular format was a short summary (1–4 pages), followed by infographics (see Appendix A for details). This formative work guided our study design and we created summaries and infographics to present information about use of walking and cycling infrastructure, examples of good cycling infrastructure, and economic impacts of new walking and cycling infrastructure in the form of benefit-cost ratios (see Fig. 1, Fig. 2 and Appendix B).

Semi-structured interviews involved three stages. The first stage involved discussion about how case study examples were normally used to influence decision-making and how they may or may not be useful. The second stage used selected case study examples from Sustrans’ Connect2 programme of new walking and cycling infrastructure as discussion prompts to investigate particular contextual factors that may or may not be useful to decision-makers (details about the Connect2 programme are published elsewhere (Le Gouais et al., 2021; Ogilvie et al., 2012)). The summary sheets for each scheme included a map of the route, changes in pedestrians, cyclists and sub-groups of users (women, female cyclists, older people, disabled/people with a long-term illness, people living in the most deprived Index of Multiple Deprivation (IMD) quintile (Ministry of Housing Communities and Local Government, 2015) and peak time users), and some local government area information (car ownership, level of commuter cycling from the 2011 census, potential for cycling from the Propensity to Cycle Tool (Lovelace et al., 2017) and IMD quintile (Ministry of Housing Communities and Local Government, 2015)). An example is shown in Fig. 1.

The final stage used a summary of results from the Connect2 programme (published elsewhere (Le Gouais et al., 2021)) that investigated associations between context and use to discuss whether these may be influential to decision-makers. The summary sheet showed associations between contextual/scheme characteristics and doubling, or increasing users by at least 50%, or very high benefit-cost ratios (≥4) (see Fig. 2).

This final stage sought to investigate views on aggregate findings compared to individual case studies, benefit-cost ratios, sub-groups of users (which may impact on inequality or congestion), and relative compared to absolute changes in users.

ALG conducted each interview in March 2020, either face-to-face, or via Zoom. A pilot interview was conducted with a colleague familiar with the local government context, to test the interview guide and case study example information sheets, ensuring there was sufficient time for in-depth discussion. Where available, ALG re-read the relevant transcript from the previous study (Le Gouais et al., 2020a) and reviewed the questionnaire feedback prior to the interviews. Interviews took an average of 63 min each (range 55–75 min). The case study example sheets and summary of Connect2 study findings were sent to participants in advance, along with the participant information sheet and consent form, and hard copies were also available for discussion in the face-to-face interviews. For interviews conducted via Zoom the prompt sheets were screen-shared with participants. The interview guide is included in Appendix C. All interviews were audio-recorded (for both face-to-face and Zoom interviews) and transcribed verbatim by a third-party transcription company; ALG checked and anonymised all transcripts before analysis. ALG also took notes during and after each interview to record any non-verbal issues which could inform the analysis.

Informed consent was obtained for all interviewees and ethical approval was granted by the University of Cambridge, School of the Humanities and Social Sciences, on 11^th^ March 2020 (Reference: 20/243).

### 2.3 Data analysis

We followed a thematic analysis method (Patton, 2002), similar to Braun and Clarke's pragmatic ‘codebook’ approach (Braun and Clarke, 2019), which follows a reflexive paradigm. This enabled us to focus on answering specific research questions whilst also allowing for flexibility to investigate emerging issues which were not identified a priori. ALG conducted line-by-line coding of all interview transcripts using qualitative analysis software NVivo 12 (QSR International Pty Ltd, 2018). Throughout the coding, ALG kept an ideas log and grouped codes into categories to order them into topic areas. These were re-categorised during the coding process which included merging and adding codes as necessary. Coding was based around the research questions but was also done inductively to allow for emerging issues and concepts to be captured. Domain summaries were produced by ALG as an early stage in the analysis around four topics: Why good examples are lacking; Important contextual factors; Showing impact; and Impact on sub-groups. Iterative discussions to develop higher-level themes were conducted between ALG , CG and LF. This went beyond summarising what was said in the interviews to gain deeper understanding about how context is considered, valued and used.

## 3 Findings

We identified three main themes from the data in this study: using examples to provide, what we called, ‘believable stories’; issues about politicised stories; and the challenges of demonstrating ‘believable’ outcomes.

### 3.1 Believable stories

Case study examples could be used to provide stories that appeared ‘believable’ to decision-makers, as well as to the public, who might be sceptical of the need to build new walking and cycling infrastructure. For these to be ‘believable’, they may need to have similar physical and socio-economic attributes and legal frameworks, be practical, and preferably be located nearby.

#### 3.1.1 Physical, socio-economic and legal contexts

Hilliness, ease of driving and car parking, and level of bus and taxi use were perceived as issues that could influence the relevance of examples because they affected the attractiveness of walking and cycling. Judging case studies' similarity to other contexts went beyond physical attributes and included social environments and social deprivation. Socio-economic demographics were described as very important because level of affluence or deprivation could influence land values and therefore the amount of money a local government was able to obtain from developers in planning obligations (‘S106’ agreements (Local Government Association, 2020)), which could be used for walking and cycling infrastructure. Local governments in more deprived areas were also likely to have fewer resources to improve the quality of built environments.

“… so in [local government area] and in [deprived region] you've got standard house builders, you're not getting world winning architects submitting schemes … For the most part you're getting an architectural technician doing it. [Local government area] doesn't have any on-site urban design advice … [Affluent local government area] has a team of urban designers, landscape architects, conservation officers. They have the professional expertise … in the Council, to be able to push back against developers. [local government area] doesn't have that.” – Local government urban planner, Area 2

Some interviewees appeared to believe that people living in deprived communities may have a stigma concerning cycling because it could appear that they were too poor to own a car. This perceived rationale for deciding whether to cycle appeared to justify a political rationale whether to fund cycling infrastructure. However, the presented examples challenged this assumption as they demonstrated an increase in use by people from deprived areas with new walking and cycling infrastructure.

“… you know full well it's lifestyle, too many chips, smoking, sedentary lifestyle, possibly pollution as well … It's difficult isn't it?.. Because a bike to them's seen as well ‘you're probably not very cool’, you know, ‘you're not, you're probably pretty poor’. Whereas if you're in [more affluent village] … cyclists are someone who's okay … It's a stigma, yeah, because people think you're poor, you know. You haven't got a car. You can't afford a car. In [more affluent village] it's a choice … And I suppose as politicians what do you gain from … giving people a multi-million pound cycle route when they don't want it?” – Councillor, Area 2

The importance of social environments shaping local physical environments also appeared to influence believability of case studies from elsewhere - best practice examples appeared less believable where the gap between best practice and the local situation was very wide. Interviewees discussed how infrastructure from places like Cambridge or Bristol with cycling cultures, and relatively comprehensive cycling networks, may not appear transferable.

“… people say, 'This isn't Cambridge, or this isn't Bristol. You know, we like our cars, we drive.' And the amount of time I've sat in public consultations and had that thrown at me.” – Private sector transport planner

Examples from other countries were said to be challenging to use because of legislative differences and the ability of international designs to pass safety audit requirements, as well as requirements to blend in with existing environments.

“… because you've used case studies from the UK they're more likely to be persuasive in influencing the planning environment than using the … Everyone knows about the Dutch and the German schemes but I think that we … know little about the impact of English schemes. So culturally I think they're probably more persuasive than saying, ‘Hey look this is what they do in Germany and this is what they do in Holland.’” – Local government urban planner, Area 1

#### 3.1.2 Solutions for local challenges

Case study examples appeared influential as believable stories if they pointed to pragmatic solutions for local challenges. For example, one developer talked about a case study example that involved a path that was narrower than the normal minimum standard. They thought this example could help in discussions with local governments where the minimum standard width appeared infeasible within the available space. Interviewees also discussed learning from poor local examples, including developments built decades previously, as well as recent examples to learn about delivery challenges, such as avoiding disjointed provision of cycling routes between parcels of land being developed by different housebuilders on a large site.

A clear justification of why walking and cycling infrastructure was built in each case study was very important to demonstrate what problem it was addressing. Some routes had been built to tackle perceived safety issues, to improve connectivity, or for leisure. Explaining why walking and cycling infrastructure was being asked for was pointed out as potentially important for developers who might see it as “one of those nice-to-haves … not an essential” (Local government transport planner, Area 1). One councillor thought that the case studies with simpler, linear routes were easier to understand than more complicated networks and therefore might be more inclined to use these types of examples.

**“**… just from my own impression, the simpler the better, so the ones that are basically straight lines … To me I think that, you know, without more information they would appeal more to me if I was trying to make the case, than the sort of bifurcated ones and indeed the network one.” – Councillor, Area 1

Although one urban planner thought that examples themselves were less important than good design principles, because they would always be adapted to a local situation, others said that the overall research presented from the Connect2 evaluation (Le Gouais et al., 2021) “tells a very powerful story” and an individual case study “brings it more alive” (Local government urban planner, Area 1). These were thought to be very useful to show to sceptical members of the public who may not think that new walking and cycling infrastructure was necessary.

“Seeing the data will definitely help [people] to understand the impact and the benefits. Sell it as a benefit, which is what people want, it's like ‘what's in it for me?’ Yeah, that is the question. If you can answer that for people you will get them on board.” Councillor 2, Area 1.

Although there were features that appeared to increase the likelihood that stories were considered believable, ultimately these seemed dependent on individual judgement and values, rather than particular objective attributes.

Interviewees grappled with the issue that there was a general lack of monitoring of new walking and cycling routes which resulted in difficulty finding suitable examples of routes in different contexts. Instead, famous locations were often used to demonstrate new walking and cycling infrastructure, including international examples, such as Freiburg in Germany for pedestrian areas, and the Netherlands for cycling infrastructure. But whilst some interviewees talked about looking at examples from mainland Europe (the Netherlands, Denmark, Sweden and Germany), this was said to be met with scepticism by councillors because “people trust local, they trust what they know” (Private sector urban planner). More so, case examples would be considered even stronger by councillors if they could visit these examples in person to observe how they worked.

### 3.2 Politicised stories

Case study examples could be judged ‘believable’ for the reasons outlined earlier, but this judgement could also be influenced by political ideology and values. The starkest example of this, clearly related to the timing of this research, was the withdrawal of the UK from the European Union (‘Brexit’), but local and national party politics featured in interviewees' accounts.

Many interviewees discussed how political issues would influence whether an example was likely to be considered by councillors, who were described as ‘tribal’ (Local government transport planner, Area 1). This was because they were reportedly less likely to consider an example from a local government controlled by a different political party. Some interviewees said that this was particularly true of more controversial projects, which could include cycling infrastructure, but it was also discussed in relation to other transport programmes, such as congestion charging.

“… members are ultimately the ones that we have to try and convince of things … And sometimes, if they're seeing schemes that are, if you're in a Conservative-led council, and all the examples we're showing them are Labour councils, they will say no just on the principle of the fact that they're Labour councils.” – Local government urban planner, Area 2

This hostility between local governments run by different political parties was also described as a problem between tiers of government which could influence infrastructure spending decisions - one councillor in Area 1 talked about a higher level of government “making life difficult” for the lower level local government, which was run by another political party.

One councillor talked about Brexit as one reason why people were reluctant to look at examples from other European countries. They said that England examples would be more positively received because they avoided Eurosceptic concerns.

“… it's interesting that your examples are from England, because obviously we're regularly told, ‘Oh well Copenhagen you know, look what they're doing in the Netherlands, and the proportion of people cycling is 40% or something, and so on’, and now that tends not to work, but partly because of course Europe is a big no-no [with Brexit] …” – Councillor 1, Area 1

Interviewees said that generally there was not a lack of support for walking and cycling infrastructure, but that the funding was limited, and money was spent on other things, suggesting it was a political decision. Many of the interviewees asked about the sources of funds for the Connect2 examples presented (e.g. from local government funds, S106 developer contributions (Local Government Association, 2020), or external funding bids) to pay for both the capital and on-going maintenance costs, as funding was often challenging to obtain.

“People do want to invest, they just haven't got the money and they have to prioritise other things … it's just central Government funding, local [governments] have got so much pressure on them now to do so much more with less, that cycling and walking kind of fall off the radar because the people that are shouting the loudest are the people that have got the giant potholes outside their road, or, there's the adult and social funding budgets come from the same pot and there's always an overspend. So all those like nice projects that the Councils really want to do, they haven't then got funding for.” - Local government urban planner, Area 2.

However, some councillors reportedly were not persuaded that walking and cycling infrastructure was needed, particularly in places where existing routes were not well used, or where it risked antagonising the car driving public who vote for them.

“… I'm thinking from a member perspective, that the push-back that you get for things around, you know, 'Ah, well, that's alright there, isn't it, but it's not what people like in [local government area] …' … They see their local population and then they're trying to think about, 'Okay, well, actually, what's this going to equate to me in votes?' [Laughs] 'Does this then save my political seat going forwards?'" – Local government urban planner, Area 2

### 3.3 Believable outcomes

Stories were more likely believable if similar and locally relevant, but the outcomes of case study examples could also influence believability because of the methods and metrics used and whether these were accepted by particular audiences.

Some interviewees were frustrated with transport modelling methods that focused on historic traffic data, making it very difficult to design for increased cycling mode share because predicted congestion called for additional road capacity, which could facilitate growth in car use. Number of vehicles, travel times and air quality were described by a private sector transport planner as “traditional metrics” but health and wellbeing were issues that they were “wrestling with” because methods needed to be “transparent” and “watertight” to be able to stand up in court if a planning application went to appeal (Private sector transport planner).

“… if we're promoting the 3000 houses and we have cited this [Connect2 example], which looks, on the face of it, as a really good proxy, there will be clever QCs who will be picking over all the evidence we're using and saying, 'Well, you've cited the [Connect2] example, which is fine, but actually we've dug into that and we feel there's, you know, flaws in the data,' or whatever they might say. Suddenly the case kind of collapses. … we're in development planning and that's quite sort of antagonistic … So anything we put into a technical document that is citing evidence, you know, in theory, we need to be entirely comfortable that we can defend that.” – Private sector transport planner.

There was a disparity between demand for ‘watertight’ evidence of impact from the development sector, which traditional transport modelling approaches claimed to provide using precedent traffic counts and which was commonly used in planning applications, and the uncertainty of health impacts and benefit-cost ratios. There was some scepticism voiced by a councillor who did not think that benefit-cost ratios were believable “because they are difficult to prove” (Councillor, Area 2). However, some interviewees thought that benefit-cost ratios were useful for local government decision-making (although not for developer contributions), and possibly that additional elements could also be included, such as economic benefits associated with improved connectivity.

“The [benefit-] cost ratio is really handy specifically where you're looking at trying to talk to members about them investing in terms of their capital programme. That is really good.” – Local government urban planner, Area 2

Some interviewees said that the case studies and research summaries presented as interview prompts (see Fig. 1, Fig. 2) were useful as evidence to demonstrate how new walking and cycling routes could be used to try to influence local transport policies, request developer contributions and use in public consultations.

“[Local government transport planner] has to fight, you know, tooth and nail to get money for the cycle and footpath infrastructure … By gathering this kind of data we're actually giving them the evidence to say that we're, you know, we're making our residents or our population healthier by creating environments that promote that, we're reducing car usage, you know, we're freeing up the roads, we're addressing air quality issues as well as, you know, obesity and other chronic lifestyle illnesses related to inactivity.” – Local government urban planner, Area 1.

Lack of clarity about the long-term impact of environmental changes appeared to challenge believability of stories. This was voiced by a public health interviewee who was not confident that use would continue over time, particularly if maintenance was not done. Another interviewee queried the impact of other concurrent interventions, such as behaviour change interventions.

Understanding the types of users of walking and cycling infrastructure was of interest to some interviewees to increase believability of a story to particular audiences, whilst others questioned the likely health impacts of new routes, for example one urban planning interviewee questioned whether the least active were likely to be gaining through use of the new routes. However, development planning decision-makers appeared to not consider the type of user to be important. Instead they appeared to value mode share because this could influence levels of traffic congestion which could be a planning constraint.

“… it's more about peak-time users and it's ways of trying to get traffic off the road to facilitate your growth in houses and potentially your growth in jobs. And so therefore, it kind of matters less, you know, who is actually migrating off the road. It's more there is one less car on the road because we put this cycle link in and, therefore, it gives us some head room in which to grow into.” – Private sector transport planner

Some local government interviewees, who were supportive of building new walking and cycling infrastructure, thought that data on relative changes in users could support the believability of a story because the outcome could easily be translated to other places, including where baseline levels of walking and cycling were low, including in rural areas.

## 4 Discussion

### 4.1 Main findings of this study

We found that case study examples could be used as stories to influence decisions on how to shape local environments, but only if they appeared believable. Case studies are likely to be deemed believable if they are similar and locally relevant, but this judgement can be inherently political and politicised and based on values. The methods and metrics used to demonstrate the impact of case studies could also affect their believability, and measures traditionally used in non-health sectors may make it challenging to create healthy environments.

Alongside specific case study examples, we found that aggregated data from multiple case studies (see Fig. 2) were perceived as useful by stakeholders to demonstrate overall value of new walking and cycling interventions. This points to the value of conducting studies across multiple locations that can then be synthesised, as done with the Connect2 study (Le Gouais et al., 2021).

**Fig. 3:**
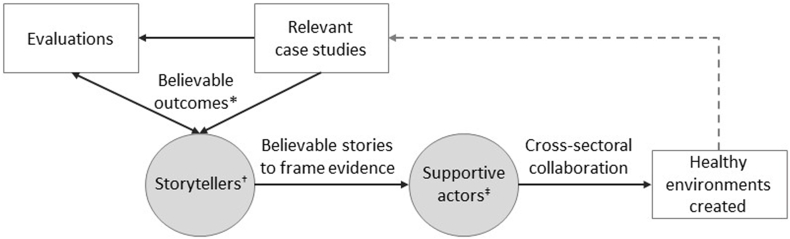
Summary of findings to create healthier environments * 'Believable outcomes’ are data or evidence that is valued by particular stakeholders, or audiences, that demonstrate certain impacts of the environment. ^†^ ‘Storytellers’ are influential knowledge brokers who can identify appropriate evidence to demonstrate impact of infrastructure change to particular audiences. They provide a credible narrative to frame evidence and explain a believable story. ^‡^ ‘Supportive actors’ are stakeholders who use the evidence, or stories, to support the creation of healthy environments.

Although influencing the creation of healthy environments is a complex issue, Fig. 3 shows a simplified view of features that appear to be important, which were identified in this study. We describe the findings within a storytelling analogy. Fig. 3 includes ‘believable outcomes’, which are data or evidence that is valued by particular stakeholders that demonstrate certain impacts of the environment. These are needed to support ‘believable stories’, which are credible narratives that frame evidence. Our interpretation supports a concept of ‘storytellers’, or knowledge brokers (Kingdon, 2010), who understand specific audiences, and how they value types of evidence about impact from case study examples. They can help to share believable stories to build support for creation of healthy environments. Greater monitoring and evaluation could provide more examples from locations that are perceived to be physically, socio-economically, and politically acceptable - depicted in Fig. 3 as a dotted line from ‘Healthy environments created’ to ‘Relevant case studies’. In the storytelling analogy greater access to case study examples across contexts can be considered as more books available in a library so that relevant stories can be found for a particular audience.

These issues are described in more detail in the following sections.

#### 4.1.1 Story settings

A case study can not only be a ‘practical example’ but also a believable story to influence decision-making. In this study we explored different elements of context, and our findings lead us to describe these within a storytelling analogy as the *setting* of a story, which can include physical, cultural, social, economic, historical, and political factors (Craig et al., 2018). Whilst each element can influence whether a case study's setting is believable enough for it to appear relevant, the issues about political context identified in this study were particularly insightful. The importance of political support to enable creation of healthy environments is not a new concept (Craig et al., 2018; Guglielmin et al., 2018; Lge-Elegbede et al., 2020; Lorenc et al., 2014; Pucher et al., 2010; Van Der Graaf et al., 2017). Political decision-makers needed to consider the schemes they supported to fit their political agendas, and be mindful of decisions that could affect election results (for example antagonising the car-driving public). Moreover, however, we found that what was considered acceptable case study settings were viewed through party political lenses. Political control of local government could be important when considering case study examples from other places, as well as being relevant for other political issues, such as about Brexit. This politicised context was highlighted in relation to whether health-promoting environments were likely to be created, because it could affect whether a positive story from elsewhere was believed, rather than necessarily affecting the transferability of interventions and their outcomes.

#### 4.1.2 Audience values

Stories can be used as tools for influence in political advocacy (Davidson, 2017) and research has highlighted that decisions can be value-driven, based on emotions, rather than scientific evidence (Cairney and Kwiatkowski, 2017; Cairney and Oliver, 2017; Knaggård, 2015).

Cairney and Oliver (2017) say that policy-makers tend to base judgments on existing beliefs, but we suggest that demonstrating the impact of new walking and cycling infrastructure on particular groups may help to tackle existing assumptions about the value of new walking and cycling routes. We suggest that this could help to build emotional connections to a story for particular audiences, which could be aided by personally verifiable case studies that can be visited locally.

Demonstrating impact for certain groups could also help tackle a singular focus on individual agency as barriers for healthy behaviours, such as walking and cycling, which seemed to be the perception held by some interviewees who discussed stigma and low social acceptability of cycling in low-income areas as reasons people did not cycle, rather than because of the quality of the infrastructure (despite evidence presented in the case study examples). This can relate to ‘victim blaming’ (Laverack, 2018), whereby unhealthy lifestyles are viewed as a choice, rather than associated with environmental factors. Connecting people through emotive issues associated with widely held values, such as fairness, is also recommended within ‘health in all policies’ guidance for local government (Local Government Association, 2016). However, we highlight that the transport sector is unlikely to consider the type of people who walk and cycle on new routes because of the metrics that are valued by this group, which could inadvertently increase inequalities, for example if new walking and cycling routes are only provided in more affluent areas as commuter routes. Rather, to achieve greater public health benefits, convenient, safe and attractive routes for multiple purposes should be provided to attract wider segments of society, including older people and those living in the most deprived areas (Le Gouais et al., 2021; Panter et al., 2019).

#### 4.1.3 Storytellers

A storyteller, often called a knowledge broker in policy analysis (Kingdon, 2010), may be necessary to help understand an audience's values and priorities and choose believable stories to share with decision-makers. Appealing to emotions and values are advocated since purely rational, evidence-based decision-making is unlikely to occur (Cairney and Oliver, 2017; Davidson, 2017; Fadlallah et al., 2019) and research has shown that urban development policy in the UK has been influenced by both evidence and ideological factors (Davoudi, 2006). Storytellers can also act as problem-brokers, highlighting issues with the status quo about the built environment (Knaggård, 2015; Le Gouais et al., 2020a). Decision-makers themselves can also become storytellers by using case study examples to justify decisions post hoc (Le Gouais et al., 2020a). Negative case studies could be cherry-picked to justify not investing in environmental interventions, but they could also be learnt from and used to justify why higher quality investments are needed so that ineffective variants are not adopted.

#### 4.1.4 Storylines

It appeared that simpler stories could be preferred by some stakeholders, such as demonstrating impact from a linear walking and cycling route, rather than from a network of routes. Although the value of simple stories has previously been highlighted (Cairney and Kwiatkowski, 2017; Cairney and Oliver, 2017; Fazli et al., 2017), evidence suggests that more connected walking and cycling networks may increase active travel (Buehler and Dill, 2015; Cerin et al., 2017; Kärmeniemi et al., 2018; Sun et al., 2014), therefore there could be a tension between simple, believable stories and impactful outcomes. Framing the overall narrative in terms of key functions, such as increasing connectivity, could help to overcome such issues (Ogilvie et al., 2020).

Cycling infrastructure in particular European countries, such as the Netherlands, is often lauded as means to achieve high levels of cycling (there are even infrastructure programmes in London that are referred to as ‘mini-Hollands’ (Aldred et al., 2019)), but this study has demonstrated some reluctance to use examples from other countries where differences are very great, making examples appear unrealistic. Cycling proponents appeared more likely to believe positive stories across different contexts, whereas sceptics needed more similarities in terms of setting and plot for a believable story. This is important since international guidance, such as from the World Health Organization (World Health Organization Europe, 2012), often highlight international examples (Jakab et al., 2018), but it may be appropriate for individual countries to depict their own ‘good practice’ examples, rather than using international ‘best practice’, to avoid legislative and ideological differences that restrict believability.

The political ideological differences, which contributed to the limited acceptability of looking to examples from other countries, may have been particularly prominent in this study because stakeholders were from England, a country that has only recently left the European Union, and this reluctance to look to other European countries may be different in other places. Other research has also found that perceptions about another country can affect its acceptability as somewhere to take ideas and learning from (Le Gouais et al., 2020a; Wood, 2015), supporting the hypothesis that good examples are not sufficient – the country itself also needs to be perceived as aspirational to decision-makers.

In complex, interdisciplinary environmental interventions, such as the creation of new walking and cycling infrastructure, there appears to be a tension between traditional metrics that are short-term and easy to measure, such as traffic counts and air quality, and less tangible, long-term outcomes, such as population physical activity and prevalence of non-communicable diseases. The latter appears as less believable outcomes to actors in the urban development sector because they require methods that are not widely accepted. However, transport assessment methods that rely on precedent traffic data can result in self-fulfilling prophecies for road requirements (a criticism that has also been expressed within transport planning in other countries (O Davies et al., 2000)). This reluctance to consider unfamiliar metrics may reflect the siloed nature of working practices across sectors.

The tension between short- and long-term outcomes can reduce political prioritisation of funding for interventions affecting the environmental determinants of health (Dahlgren and Whitehead, 1991). This suggests a need to emphasise the short-term outcomes of these multi-disciplinary interventions, such as congestion, mode share, and safety, but could also involve emphasis of impacts on particular target groups, such as older people, to create emotive engagement, as described earlier.

Conceptually, this study may also contribute towards understandings of what might be broadly called ‘place-making’. This term is commonly used in urban planning to describe the process for creating sustainable, well-designed environments that meet people's needs and improve quality of life (Glasgow City Council, 2018). However, the concept has been used more widely in human geography and political sciences to refer to “the set of social, political and material processes by which people iteratively create and recreate the experienced geographies in which they live” (Pierce et al., 2011). We found that decision-making for place-making should also be understood as an envisioned experience – a believable vision of the kind of place we want to live in and how places could be altered for better health. We propose this as a direction for future research.

### 4.2 Strengths and limitations

This was a small study, following-on from a previous qualitative study (Le Gouais et al., 2020a) and quantitative study (Le Gouais et al., 2021) by the same lead author and colleagues. We tried to include a range of participants across different locations and from different disciplines, although we were unable to conduct repeat interviews with all participants due to resource constraints and loss of contact.

This study was conducted in England within the specific context of Brexit. Although that is a unique situation, populism and divisive politics is a feature of many different settings at the present time, and this has been seen in local objections to temporary cycle lanes in response to COVID-19. Conducting a similar study in other countries would be useful in understanding whether the reluctance to look to other countries for case study examples would be found elsewhere, including whether countries without a dominant two-party political system had similar ‘tribal’ tendencies when it came to local government decision-making. It would also be useful to test the conceptual model, shown in Fig. 3, for other environmental issues that can influence population health, both at a local level, such as restricting hot food takeaways or creating age-friendly environments, and also at national level, such as decision-making for taxation of sugar-sweetened beverages.

ALG produced the discussion aids from evaluation of the Connect2 programme (Le Gouais et al., 2021). This provided real examples for participants to engage with, to draw on their experiences and perceptions. ALG had not been involved in the original Connect2 programme or its original independent scientific evaluation, but had conducted a further evaluation on which the interview prompt sheets were based as part of a mixed methods PhD (Le Gouais, 2020). Whilst she did not emphasise her role in that work to participants, it may be that people responded positively to her because they were familiar with her and therefore acted courteously which may have influenced their responses. She shared findings of the previous research with participants (Le Gouais et al., 2020a) which may have influenced their assumptions about what this study was about, and subsequent discussions.

We recognise that ALG, in sharing the case study examples in the interviews, took on the role of storyteller, and in fact some interviewees asked if they could share the summary sheets with colleagues following the interviews (versions with organisational logos were provided later for this purpose). However, the motivation for this research was not to present ‘believable stories’ to stakeholders, but to understand what it was about the examples that might appeal or not. Still, the active role of the researcher should be acknowledged (Braun and Clarke, 2019) and we recognise that had this study been conducted by other researchers they may have developed different findings.

## 5 Conclusion

In our study of the relevance of case studies for influencing decision-making we demonstrated the importance of telling believable stories. Case studies can go beyond being ‘practical examples' and can have the power to influence decision-making, but they need to be similar and locally relevant, and the onus is on the storyteller to highlight these similarities. Organising and framing multiple pieces of evidence (studies or outcomes) into believable stories that make local contextual sense can help to convince decision-makers.

We found that believable stories could be used to increase acceptability of environmental changes to support walking and cycling by local people and decision-makers. Examples from England were generally preferred to those from abroad, particularly because of Eurosceptic attitudes which restricted emotional connections to international stories. Physical and socio-economic similarities could be necessary, but not sufficient, conditions for story settings to appear believable, whereas local party politics could affect acceptability of using examples from other local government areas. Clarity of purpose of individual examples was also important to define the plotline of a story. The need for ‘watertight’ calculations in transport assessments made it difficult to design for high levels of active travel in new developments or to incorporate health and wellbeing metrics, as these outcomes were less believable to some audiences.

Greater availability of case study examples across contexts could help increase the library of stories available for storytellers to choose from. This could require greater monitoring and evaluation in different physical, socio-economic and political contexts, as well as demonstrating impacts on different types of people, such as older people and those living in deprived areas. This could strengthen emotional engagement with believable stories for relevant audiences to facilitate investment in healthy environments.

## What makes a case study example ‘believable’?

• Similar enough to appear relevant in terms of physical and demographic features.
• Politically palatable, e.g. brought in by the ‘right’ political party, following the party line or agenda, speaking to constituency values.
• Useful enough and close enough to home to directly address local issues.
• Easy enough to be understood and engaging (brought to life).

## Funding

ALG, DO and JP were supported by the Medical Research Council [grant number MC_UU_12015/6 and MC_UU_00006/7]. The work was undertaken by the Centre for Diet and Activity Research (CEDAR), a UKCRC Public Health Research Centre of Excellence. Funding from the British Heart Foundation, Cancer Research UK, Economic and Social Research Council, Medical Research Council, the National Institute for Health Research, and the Wellcome Trust, under the auspices of the UK Clinical Research Collaboration, is gratefully acknowledged.

LF was funded by the National Institute for Health Research (NIHR) (16/137/64) using UK aid from the UK Government to support global health research. The views expressed in this publication are those of the author(s) and not necessarily those of the NIHR or the UK Department of Health and Social Care.

No funder had any role in the study design; data collection, analysis, or interpretation; in the writing of the report; or in the decision to submit the article for publication.

## Declaration of competing interest

None.

## Figures and Tables

**Table A.1:** Feedback questionnaire: Usefulness of additional information. Q1. How useful would further evidence summaries on these topics be for you?

Topic	Very Useful	Useful	Unsure	Of Little Use	Not Useful
1. Health benefits of physical activity, including walking and cycling					
2. Health benefits of green or open space					
3. Health impact of living in different environments/place-making					
4. Use of new walking and cycling routes					
5. Economic impacts of walking and cycling routes and other place-making					
6. Examples of good cycling infrastructure and other ALI designs					

**Table A.2:** Feedback questionnaire: Format of information. Q2: How likely is it that you would engage with the following formats?

Type of resource	Very Likely	Likely	Unsure	Unlikely	Definitely Not
Report (10–40+ pages)					
Short summary (1–4 pages)					
Academic research article					
Podcast (5–10 min)					
Infographic					
Other (please specify)					

**Table A.3:** Questionnaire respondents by local government area

Area	Total contacted	Total responses (%)
1	19	12 (63)
2	9	4 (44)
3∗	15	8 (53)

∗ Participants from Area 3 were not included in this follow-up study because this allowed a greater range of stakeholders from the two other areas to be included. These two areas had the greatest contextual differences between each other (level of deprivation/affluence, urban/rural, cycling infrastructure etc.).

**Fig. A.1:**
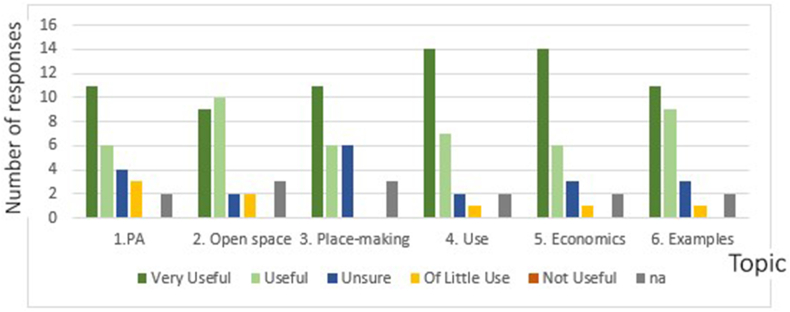
Summary of feedback about usefulness of identified topics.^1^.

**Fig. A.2:**
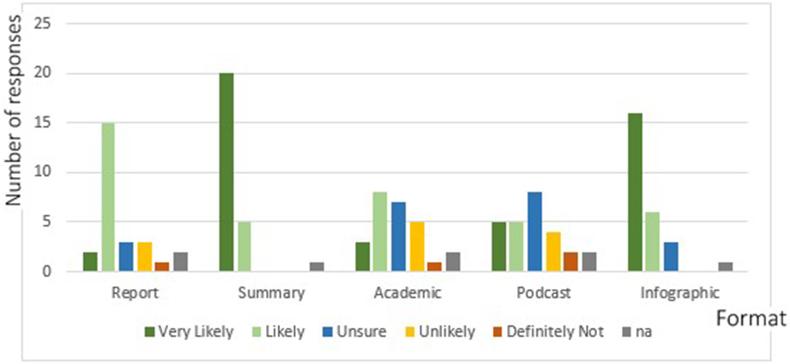
Summary of feedback about likely engagement with different formats of information.^2^.

**Table A.4:** Case study examples used in interviews with participants from each local government area

Scheme	Local government area	Topography	Estimated baseline cycling*	% change cycling	Estimated baseline walking*	% change walking	Cost (£)	Length (km)	IMD quintile (1 = most deprived)	Percentage of cycling (PCT)^†^	Potential for cycling (PCT)^‡^	Benefit-cost ratio
**Area 1 (semi-rural)**		**Flat**	**-**	**-**	**-**	**-**	**-**	**-**	**5**	> **5**	**19**	–
Carlton-Le-Moorland – Bassingham link	North Kesteven	Mixed	10,019	136.2	35,910	53.8	502,000	2.1	5	4.3	19.0	5.4
Kenilworth – Burton Green greenway and link to the University of Warwick	Warwick	Mixed	8159	767.1	62,475	195.5	1,153,000	9.9	5	3.5	20.3	10.9
Leicestershire: Watermead Park links	Charnwood	Mixed	67,285	42.4	363,671	40.6	1,691,000	7.8	4	4.1	20.8	8.1
Nantwich – Crewe link	Cheshire East	Mixed	42,626	43.5	67,396	60.1	1,560,000	6.3	5	2.9	17.8	4.0
Sleaford – Leasingham link	East Hampshire	Mixed	34,597	55.7	306,832	76.0	871,000	2.6	5	1.9	11.6	3.7
Worcester links and canal towpath	Worcester	Mixed	168,629	23.6	1,926,199	62.9	4,427,000	17.1	3	4.6	22.5	30.8
**Area 2 (urban with regeneration)**		**Mixed**	**-**	**-**	**-**	**-**	**-**	**-**	**2**	< **3**	**16**	–
Bethnal Green local link	Tower Hamlets	Flat	32,917	49.7	234,513	128.1	2,244,000	2.9	1	7.0	30.6	9.0
Blyth network	Northumberland	Hilly	51,224	68.1	609,925	11.9	2,503,000	14.5	3	1.6	14.6	3.5
Croydon parks links, crossing duel carriageways	Croydon	Mixed	15,140	95.0	315,421	273.5	1,868,000	2.3	2	1.3	16.3	16.1
Dover greenway to city centre and seafront	Dover	Mixed	11,368	95.9	543,678	45.5	757,000	2.8	2	2.3	13.4	22.3
Nantwich – Crewe link	Cheshire East	Mixed	42,626	43.5	67,396	60.1	1,560,000	6.3	5	2.9	17.8	4.0
Plymouth network	Plymouth	Hilly	110,247	23.1	672,637	62.9	2,090,000	10.9	2	2.8	13.2	9.2
Tyne Dock safety improvements	South Tyneside	Mixed	68,441	45.6	61,002	−0.1	586,000	1.6	1	2.3	22.0	7.6

*Estimated baseline levels of walking and cycling for each scheme were found by Sustrans using the method described elsewhere (Le Gouais et al., 2021).

† An indication of percentage cycling for the study local government areas are shown, to reduce likelihood of area identification.

‡ Percentage potential for cycling is rounded for the study local government areas to reduce likelihood of area identification.

**Fig. B.1:**
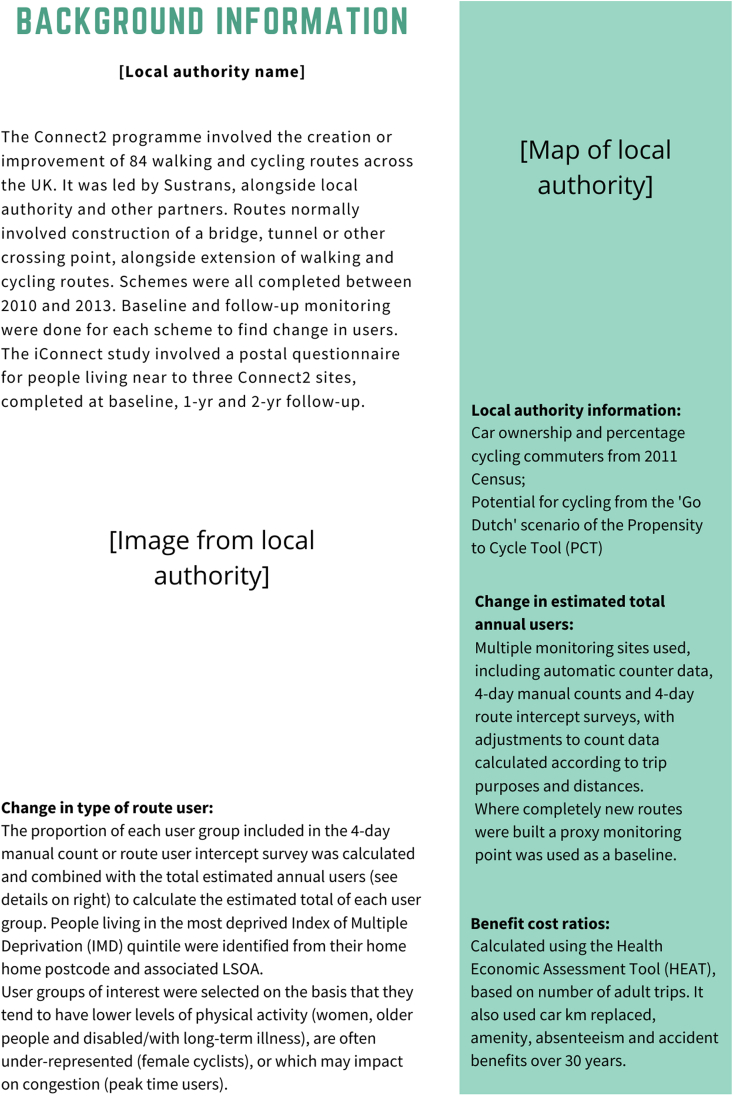
Example Local government background information sheet.

**Table:** 

No.	Main question	Possible follow-up
1	Can you explain if and how you use examples of walking and cycling routes to inform decisions about new walking and cycling infrastructure here?	• Do you have any examples of when they influenced decision-making for new walking and cycling infrastructure?
**2**	**Where** do you get case study examples from?	• Are they from this local government area or elsewhere? • How do you find out about them? • Do you use any of the **resources** I previously sent links to? [show links to resources sheet]

**Table:** 

No.	Main question	Possible follow-up
**3**	What **contextual features** or route characteristics do you think are **most and least important** to make them useful as examples to you?	• Discuss some contextual factors which are clear in the **examples**: Rural/urban; topography; percentage cyclists; car ownership; level of deprivation. • What else would be useful to know about? Prompt about social/political issues if only physical environmental issues mentioned.
**4**	Is the information about how **different groups** of people may use the routes useful?	• How much might you consider **inequalities** when influencing decisions about new walking and cycling infrastructure?
**5**	Can you comment on the information about **benefit cost ratios**?	• Do you think it is useful? How/why? Or why not?

**Table:** 

No.	Main question	Possible follow-up
**6**	Do you think these types of overall findings can be useful in decision-making for new walking and cycling routes?	• What would be more influential: doubling users or having a very high BCR? • Is the information about peak time users useful?
**7**	Can you comment on the usefulness of absolute and relative changes in users?	• Are infrastructure decisions here influenced by the government's target to double the number of cyclists? • Prompt that low baseline may be easier to double than high baseline.

## Appendix A Participant feedback

### Gap analysis

We sought to provide the original study participants (Le Gouais et al., 2020a) with additional information or evidence that might be useful to them, alongside feedback from that study. To identify these potential issues, we conducted a gap analysis of original study stakeholder interviews involving line-by-line coding of transcripts, supported using NVivo12. We identified issues where three or more participants appeared to lack knowledge or understanding, or explicitly sought additional information. This resulted in six topics, as shown in Table A.1. These included: use of new walking and cycling routes; economic impacts of walking and cycling routes; and examples of good cycling infrastructure and other ALI designs.

### Obtaining participant feedback

We identified and sent links to resources related to the topic areas identified in the gap analysis to all participants, plus their colleagues who had expressed interest in the study (N = 43). Participants were contacted individually by email in late October 2018 alongside a summary report.

Feedback from participants was also sought about the identified topics, as well as preferred formats of information using a short questionnaire with Likert scales (Likert, 1932), shown in Tables A.1 and A.2. This was to check the demand for each topic area by different types of stakeholders to help shape this project. The request for the questionnaire to be completed was included within the summary report, and also in the covering email, asking for it to be returned by email. Reminders were sent to participants together with an additional resource link about health and transport in December 2018.

### Results of participant feedback

Responses to the questionnaires were received from 56% of people (see Table A.3). Feedback received from participants on the usefulness of evidence summaries and formats can be seen in Figs. A.1 and A.2. There was a high level of interest for more information about all the topic areas from most of those who responded, as seen in Fig. A.1, although sample sizes were small. This included apparent demand for more examples of good cycling infrastructure and greater understanding about use of walking and cycling infrastructure - 83% and 88% of respondents respectively thought additional information and evidence about these issues would be useful or very useful. Economic impacts of new walking and cycling routes were also reported to be useful or very useful by 83% of respondents.

As seen in Fig. A.2 the most supported format was a short summary (1–4 pages), followed by infographics, which 100% and 88% of respondents, respectively, reported that they were likely or very likely to engage with. Academic research articles and podcasts were the least popular choices.

This formative work guided our study design. As described in more detail below, we used short summaries and infographics to present information to participants about use of walking and cycling infrastructure, examples of good cycling infrastructure, and economic impacts of new walking and cycling infrastructure in the form of benefit-cost ratios.

## Appendix B Interview prompts

### Selection of case study examples

We chose six schemes from the Connect2 programme for each local government area to be used as discussion prompts in the second stage of the interviews. These each had some similarities with the study areas, such as the type of rural/urban area; level of deprivation (similar Index of Multiple Deprivation quintiles for the local government (Ministry of Housing Communities and Local Government, 2015)); level of cycling (using data from the Propensity to Cycle Tool (PCT) (Lovelace et al., 2017)); and topography (particularly hilliness). The examples each had benefit-cost ratios of at least 2 (‘high’ value for money as defined by the UK's Department for Transport (2015)), and most were much higher. Schemes which saw decreases in cyclists or total users were excluded. Schemes which had unusual settings or aspects were excluded, for example if the majority of a new route was composed of a coastal path since these are quite specific contexts which may not have been relevant for the focal local government areas. Schemes in Scotland and Northern Ireland were excluded because there was no PCT data available. This resulted in short lists of 11 Connect2 schemes for each area from which six were chosen, covering a variety of types of settings. Table A.4 shows the schemes used in the interviews for each of the two local government areas. After piloting the interview in Area 1, we decided to also include one of these case studies as an additional example in Area 2 because it appeared to be a potentially useful example of a new housing development connecting to an existing town. Therefore we used six examples with interviewees from Area 1 and seven examples with interviewees from Area 2, although in some interviews our discussions focussed on only a handful of these.

### Interview prompts: summaries and infographics

The participant questionnaire feedback demonstrated preference for short summaries and infographics, therefore we used these formats to present data for discussion during the semi-structured interviews. This was to engage participants to encourage dialogue during the interviews as well as to increase the likelihood of interviewees reading the material prior to the interviews. We produced summary sheets and infographics with Canva,^3^ a free design website, to use as interview prompts. These included:

1. A summary sheet for each local government area entitled ‘Background information’, which included local government information that could be compared to the individual case study examples, such as level of commuter cycling from the 2011 census and potential for cycling from the PCT. It also outlined the methods used in evaluating the Connect2 programme. An (unidentified) example is included in Fig. B.1.
2. A sheet for each of the case study examples. These included a summary of the scheme, a map of the route, changes in pedestrians, cyclists and sub-groups of users, and some local government information. An example is shown in Fig. 1.
3. A summary of the Connect2 analysis results (described in full elsewhere (Le Gouais et al., 2021)) showing associations between contextual/scheme characteristics and doubling, or increasing users by at least 50%, or very high benefit-cost ratios (≥4). See Fig. 2.

## Appendix C: Semi-structured interview guide

### Context study - Interview guide

[Documents used as discussion aids: 1 - Links to resources; 2 - New route examples (6 for the relevant local government area); 3 – Background information; and 4 - Summary of Connect2 study results.]

**Introduction:** Purpose of the study and how it follows on from some of the findings about contextually relevant examples raised in the earlier project on decision-making for walking and cycling infrastructure and open spaces.

Stage 1: How case study examples are used and how they're accessed (including thinking back to previous interview comments and feedback about resources previously sent).

Stage 2: Discussion about usefulness of new route case study examples [up to 6] and contextual features.

I've got [six] examples of new walking and cycling routes which were built with Sustrans and local authorities as part of the Connect2 programme. I think there are some similarities between them and this local government area but I'd like to get your feedback on whether you think these examples could be useful here to influence decision-making.

[Spread out the 1-page new route examples and background information].

Stage 3: Discussion about how aggregate research findings may or may not be useful.

Our research group has been involved in evaluating the Connect2 schemes using data from Sustrans - there were 84 in total from across the UK. We have combined information across all of these schemes to get a sense of the overall picture of how much the schemes are used and who by. I'd like to get your views on whether this type of evaluation is useful to you, or how you think it could influence decision-making for new walking and cycling infrastructure.

[1-page summary of the Connect2 study results].

**Outline study findings:** Summary of the results e.g. percentage of schemes with increases in users; association between context/scheme characteristics and outcomes found from analysis.

[Allow interviewees to give unpromoted feedback on the presentation of the 1-page summaries but if necessary explain that this is not the main aim of the study, rather it is to understand whether this type of information may be useful.]

**Wrap up**:

9. Any other comments/thoughts?

Thank you for your time. I'll put together a summary of findings and share them with you, like I did with the first part of the study.

## Footnotes

1 Numbered topics correspond to the topics includes in Table A.1.

2 Formats correspond to the formats included in Table A.2.
